# Insights into Monkeypox Virus: Host Immunity, Viral Immune Evasion, Recent Advances in Vaccines, Therapeutic Development, and Future Perspectives

**DOI:** 10.3390/microorganisms14020317

**Published:** 2026-01-29

**Authors:** Mingzhuo Chen, Weigang Ren, Xin Wu, Jamal Muhammad Khan, Humera Nazir, Shafeeq Ur Rehman, Faizan Ali, Junwei Li

**Affiliations:** 1 Department of Infectious Disease, The Second Hospital of Nanjing, Affiliated to Nanjing University of Chinese Medicine, Nanjing 210003, China; 17660537595@163.com; 2 Division of Hepatobiliary and Transplantation Surgery, Department of General Surgery, Nanjing Drum Tower Hospital, The Affiliated Hospital of Nanjing University Medical School, Nanjing 210003, China; rgw20161564@163.com; 3 Yunnan Joint International R&D Center of Veterinary Public Health, College of Veterinary Medicine, Yunnan Agricultural University, Kunming 650051, China; wuxinddl@hotmail.com; 4 Department of Parasitology, Cholistan University of Veterinary and Animal Sciences, Bahawalpur 63100, Pakistan; 5 Department of Microbiology and Molecular Genetics, Bahauddin Zakariya University, Multan 60000, Pakistan; humeranazir@bzu.edu.pk; 6 Department of Microbiology, Cholistan University of Veterinary and Animal Sciences, Bahawalpur 63100, Pakistan; 2019-cu-micro-003@cuvas.edu.pk (S.U.R.); faizansarwarali@gmail.com (F.A.)

**Keywords:** Monkeypox virus (MPXV), Mpox, orthopoxvirus, diagnosis, vaccine development, therapeutic strategies, antiviral agents

## Abstract

Monkeypox (Mpox), a zoonotic viral disease caused by the Monkeypox Virus (MPXV), has gained significant attention in recent years due to its increasing incidence and the grave threat it poses to global health. MPXV has spread at a rapid pace during the COVID-19 pandemic, causing 10,000+ confirmed cases and ~300 fatalities in 122 countries. This virus comprises two major clades, Clade I (Central African), which is evidently more virulent, and Clade II (West African), which has caused the recent outbreaks across the world and caused fewer deaths. Clinically, Mpox presents as a milder form with fever, lymphadenopathy, and vesiculopustular rash similar to smallpox. Diagnostic measures such as polymerase chain reaction (PCR) are the main diagnostic confirmatory tools. Advanced diagnostics involve electronic microscopy, serology, and immunohistochemistry. Alternative drugs like tecovirimat and brincidofovir have demonstrated potential for treating smallpox, but there is scanty evidence on their efficacy against MPXV. Most recent advancements in the study of vaccines have resulted in the creation and introduction of MVA-BN (JYNNEOS/Imvanex/Imvamune) and ACAM2000 vaccines, which conferred cross-protection against MPXV. MVA-BN is suggested to perform better than other types due to its enhanced safety and immunogenicity. Researchers are also developing DNA and protein subunit vaccines against Mpox to induce specific immune responses by presenting viral proteins. The discovery of novel vaccine candidates and antiviral treatments will be needed to prevent future outbreaks and reduce the global health burden of Mpox. This review focuses on the characterization of MPXV, summarizing current knowledge on its genomic structure, pathogenesis, replication, potential targets of anti-MPXV drugs, clinical features, and epidemiological patterns, along with recent advances in vaccine development.

## 1. Introduction

In the year 2022, the World Health Organization (WHO) reported a substantial global rise in MPXV infections outside Africa during the recent post-COVID-19 pandemic, and this consistent fast-track spread of Mpox has emerged as a serious health concern. The first case of Mpox viral infection was reported to have a travel linkage from Nigeria to the United Kingdom; nonetheless, the present epidemic appears to have spread through channels beyond travelling. MPXV is a viral-zoonotic infection exhibiting characteristics comparable to those observed in the smallpox virus infection, with smallpox causing mild psychological stress compared to that of Mpox [1].

The MPXV is a member of the family Poxviridae and genus Orthopoxvirus. Poxviruses have a significant tendency to target unusual biological hosts, particularly in a population with compromised immunity [2]. This was observed in 2003, when MPXV made its debut in the Western Hemisphere, and Mpox-infected cases surged in the Midwest of the United States of America [3].

As of 11 September 2022, 57,527 confirmed mpox cases and 18 fatalities were reported from 103 countries and territories [4]. Endemic outbreaks occurred in the Central African Republic, Democratic Republic of Congo, Gabon, and Ghana. From January to December 2023, approximately 9000 new cases and 171 deaths were reported worldwide, rising to around 24,880 cases with 263 deaths in the year 2024 [5]. During 2025, the outbreak intensified from January to August, with about 38,671 cases and 163 deaths reported globally. From 14 September to 19 October 2025, an additional 2862 new cases and 17 deaths occurred (https://www.cdc.gov/monkeypox/situation-summary/index.html, accessed on 25 Occtober 2025), mostly across African countries experiencing active transmission, as shown in Table 1. A gradual increase in the number of MPXV infection cases has been reported, and with the surge of the epidemic, newer cases of MPXV infection are also being documented [6].

The MPXV genome, available in the NCBI database, reveals that the viral strains present in the recent outbreak in Africa are distinct from West African strains, inducing moderate infectivity and lower mortality [15]. MPXV consists of linear double-stranded DNA (dsDNA) with inverted terminal repeats (ITRs) forming hairpin loops, tandem repeats, and open reading frames (ORFs) at genome ends. Viral replication occurs in the cytoplasm, encoding proteins necessary for DNA replication, virion assembly, RNA expression, and immune evasion [16]. No medications or vaccines are currently available licensed specifically for human mpox. The Dryvax smallpox vaccine has been used historically against MPXV, administered via multiple skin punctures with a bifurcated needle. Long-term storage may weaken vaccine efficacy, and recipients reported adverse effects [17]. Therefore, there is a dire need for a next-generation vaccine to confer immunity against Mpox. In this regard, scientists have developed the next-generation vaccine by going through the whole repertoire of proteins encoded by the MPXV genome, which consists of 176 different protein chains. They have examined each one of the common antigenic receptors found on B cells and T cells. To develop a peptide, the next-generation vaccine against the MPXV, some common epitopes that had high antigenic scores were taken into consideration. The vaccine elicits a more rapid immune response than the infecting virus, which is the fundamental concept reinforcing all forms of vaccinations [18]. Classical vaccines are manufactured via a series of biochemical tests to produce antibodies in vaccinated individuals. In addition to being cost-ineffective, time-consuming, and allergenic, these vaccinations are also produced by a procedure that necessitates the in vitro development of infectious bacteria and raises eyebrows over safety [5]. In contrast, epitope vaccines that are manufactured based on peptides offer an exceedingly safe and cost-effective alternative to the conventional vaccine [19]. The effectiveness and safety of vaccines mainly rely on the technical requirements throughout their production, development, and deployment [20]. This manuscript adheres to the TITAN Guidelines 2025 to ensure transparency, integrity, and responsible authorship in scientific publishing [21].

This manuscript provides a distinct contribution beyond existing Mpox reviews by offering an integrated translational synthesis rather than a single-domain summary. Specifically, it links viral genomics and immune-evasion mechanisms with host innate and adaptive immunity, clinical severity, and clade-specific differences; critically evaluates post-2022 evidence including APOBEC3-associated mutations, randomized antiviral trials, and real-world vaccine performance; compares vaccine platforms at the immunological mechanism level; assesses diagnostics and therapeutics through a global-health feasibility lens; and explicitly defines unresolved knowledge gaps and future research priorities. By synthesizing developments from 2018 to 2025 into a cohesive framework connecting molecular mechanisms with translational and policy-relevant outcomes, this review offers added value beyond the previously published literature.

Several Mpox reviews published between 2023 and 2025 have provided timely and valuable updates on the re-emergence of the disease, primarily focusing on epidemiological trends, clinical manifestations, transmission routes, and general public health responses, or addressing single domains such as immune evasion, vaccines, or antiviral therapies in isolation. While these reviews have been instrumental in summarizing rapidly evolving knowledge, they often adopt a descriptive or compartmentalized approach, with limited integration of viral genomics, immune-evasion mechanisms, host innate and adaptive immune responses, and clade-specific differences in disease severity. In addition, many do not fully incorporate post-2022 genomic insights, APOBEC3-associated mutations, or recent randomized clinical trial evidence for antivirals, nor do they systematically assess diagnostics and interventions from a global-health and feasibility perspective, particularly for endemic and low-resource settings. In contrast, the present review extends beyond the existing literature by offering an integrated translational framework that connects viral evolution and immune evasion with host immunity, clinical outcomes, diagnostics, vaccine platform immunology, and therapeutic decision-making, while explicitly identifying unresolved knowledge gaps and future research priorities relevant to basic, clinical, and public-health research [22,23].

### Data Sources and Literature Search Strategy

A systematic literature search carried out in a structured manner was used to carry out this review to cover all the recent developments concerning the MPXV. PubMed, Google Scholar, Scopus, and Web of Science databases were used to retrieve relevant articles related to the research topic and published from January 2018 to September 2025. Moreover, reporting and recommendations of recognized health organizations like the World Health Organization (WHO), the Centers for Disease Control and Prevention (CDC), and the European Centre of Disease Prevention and Control (ECDC) were also investigated to supplement scientific evidence. The studies were also filtered out based on non-English publications, non-peer-reviewed preprints, and research on other Ortho poxviruses with no direct comparative relevance to MPXV. The literature identified was critically analyzed to summarize genomic features, diagnostic methods, treatment options, and vaccine developments, and outline research gaps and future perspectives. “Epidemiological data for 2023–2025 were extracted exclusively from official WHO, PAHO, and ECDC situation reports (no projections or modeled estimates were used). All 2025 case counts represent preliminary confirmed cases as published by WHO up to 30 October 2025.” As presented in Table 2, mpox can be differentiated from smallpox and chickenpox based on its distinct lesion stages and lymphadenopathy.

## 2. The Origin and Classification of MPXV

**Figure 1:**
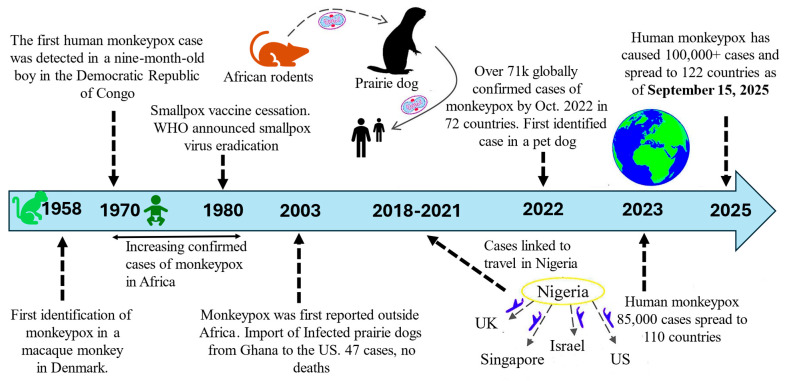
Timeline of MPXV from 1st identification until current outbreak.

**Figure 2:**
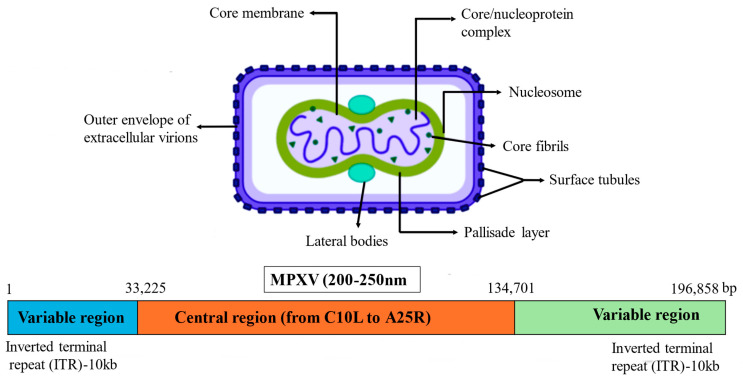
The structure of the MPXV and its genome.

There are 22 genera and 83 species that belong to the family Poxviridae. These species are divided into two subfamilies: Chordopoxvirinae, which has 18 genera and 52 species, and Entomopoxvirinae, which has 4 genera and 31 species [26,27]. Each of the twelve members of the genus Orthopoxvirus, which infects both people and animals, has been identified. The variola virus, which is responsible for smallpox, is the most well-known member of the family. Viruses belonging to the West African and Central African (Congo Basin) clades have been identified and constitute two distinct viral groups. Viruses that originate in central Africa are more lethal than those that originate in West Africa [28]. A positive association was shown between the Central African origin of human Mpox disease and higher levels of morbidity, death, human-to-human transmission, and viremia during the 2003 pandemic in the United States [29]. The timeline of MPXV from its 1st identification until the current outbreak is shown in Figure 1 and Figure 2 represents the structure of the MPXV and its genome

Experimental findings indicate that the Central African clade is more pronounced and has a higher mortality rate (10%) in comparison to the West African clade, which only shows a mortality rate of 4% [30]. Variabilities in genomic organization, which are produced by deleted gene sections and gene fragmentation in open reading frames, are the source of the variances in virulence [17]. Therefore, it is essential to collect samples from a variety of locations, people, and clades to ascertain the genetic characteristics of the MPXV and to verify the cases and research facilities [31]. The genomic and phylogenetic classification of MPXV is summarized in Table 3.

## 3. Transmission of MPXV

**Figure 3:**
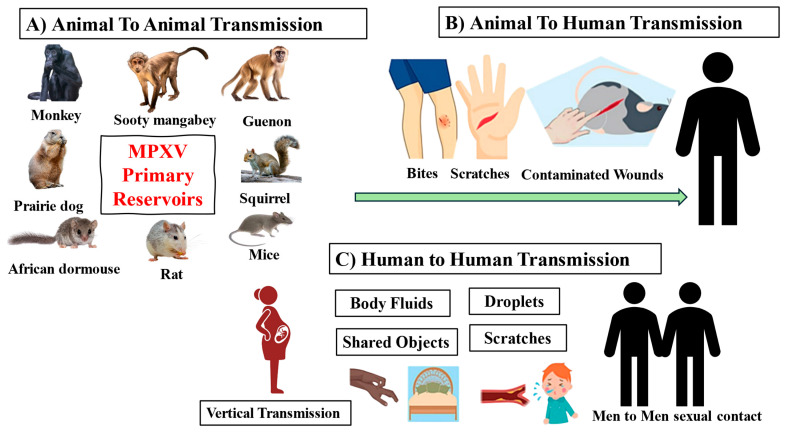
The transmission of the MPXV: (**A**) Animal-to-Animal, (**B**) Animal-to-Human, and (**C**) Human-to-Human.

West and Central Africa have been the primary locations where Mpox has been detected. The transmission of MPXV may take place in some different modalities, including animal-to-animal, animal-to-human, and human-to-human transmission (Figure 3). Direct contact with an infected animal or the contamination of body fluids is the most prevalent method of transmission from animals to humans [33]. There has been a correlation between human infections and interaction with animals. Studies undertaken in Nigeria and West Africa have shown human-to-human transmission [34]. MPXV may be transmitted to humans via intimate, personal contact, which is often skin-to-skin contact. It can also be transmitted by contact with respiratory secretions, contaminated objects, materials (clothes, bedding, or towels), and surfaces that have been used by someone who has Mpox. The placenta is the way through which the virus may be transmitted from a pregnant woman to their unborn child. It is very definite for humans to get Mpox from animals that are infected with the disease [35]. This may happen if the animal scratches or bites a person, or if the person prepares or consumes meat that comes from a diseased animal. Individuals infected with Mpox can transmit the disease to others from the moment they experience the first symptoms until the rash has completely healed, and a new layer of skin has grown. In addition, the CDC examined further documented cases of suspected transmissions, such as embracing, kissing, oral, anal, and vaginal sexual intercourse. These transmissions may be linked to genetic alterations that enable the transfer of the MPXV from one human to another [30].

The spread of Mpox and the progression of outbreaks are key factors influenced by socio-behavioral factors. Overlapping sexual connections and intimacy during recent outbreaks have influenced the spread of viruses, even in cases where the virus itself is not exceptionally virulent [36]. Stigma, inadequate health infrastructure, ineffective medication, and the rusty orthodox cultural and social norms play a crucial role in giving rise to Mpox infections in individuals who seek care, timely medication, vaccination, and authentic documentation of the cases recovered from Mpox infections. Establishment of technical and technological advanced diagnostics facilities, appointing relevant qualified healthcare officials, provision of effective medicines, and an enlightened approach to the orthodox network may address the worsening critical situation to a certain extent. Media and associated channels can and should be involved to ensure intake and induction of modern, effective therapy [37].

## 4. Genome Organization and Viral Entry Mechanism of the MPXV

The genome of MPXV is composed of double-stranded DNA (dsDNA), approximately 197.2 kilobases in length, and encodes 181 proteins. No free 3′ or 5′ ends are present in the linear genome of MPXV, which contains hairpin ends that are covalently closed. Figure 2 shows that inverted terminal repeats (ITRs), which are 10 kb in length, can be found at both ends of the genome. Intergenic regions that are greater than 100 base pairs are very rare, and genes are densely packed. The conserved core region is where the genes responsible for transcription, replication, and virion assembly are encoded [38], and these genes are referred to as “housekeeping” genes, which are different from one poxvirus to another. Proteins that are involved in illness and host range are produced by the genes that are encoded in the terminal domains of distinct poxviruses. Isolate MPXV_U.S._2022_MA001 is the first full MPXV genome of the current MPXV epidemic. It has been published in the GenBank database with an accession ID of ON563414, as of the 30 May 2022, via the website https://www.ncbi.nlm.nih.gov/nuccore/ON563414 [39].

**Figure 4:**
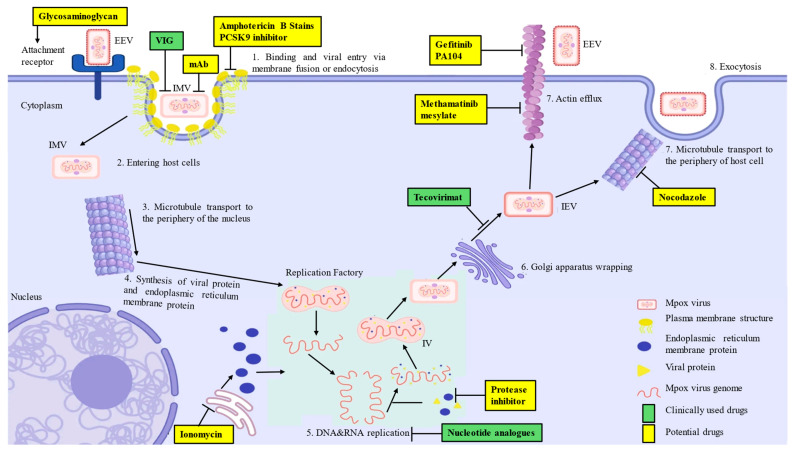
The life cycle of the MPXV, illustrating viral entry, infection, replication, assembly, and excretion within the host cell. The figure also highlights potential antiviral targets, including viral entry inhibitors, DNA replication blockers, and inhibitors of virion assembly and release. EEV—Extracellular enveloped virus, IEV—Intracellular enveloped virus, IMV—Intracellular mature virus, IV—Immature virion, VIG—Vaccinia immune globulin, mAb—Monoclonal antibody.

The size of MPXV is within the range of 200 to 250 nm, and upon infection, the MPXV undergoes replication inside the cytoplasm of the host cell. A lipoprotein envelope, double-stranded deoxyribonucleic acid (dsDNA), and lateral bodies describe the core portion of the virus [40]. Pathways such as the nasopharyngeal, oropharyngeal, subcutaneous, intradermal, and intramuscular channels facilitate the entry of viruses. Micropinocytosis, viral endocytosis, and cell membrane fusion are the mechanisms that allow viral entry [41]. After inoculation virus reproduces and sets off the process of inflammatory immune-mediated phagocytosis. This, in turn, enables the virus to move to other organs, including blood, lymph nodes, tonsils, bone marrow, and spleen. Under the direction of MPXV mature virions (MV) and enveloped virions (EV), the genome and proteins of the MPXV are released into the cells of the host [42]. The transcription and translation of the MPXV mRNA, results into the production of intracellular mature virions (IMV) containing viral DNA that encodes the virus naturally. Intracellular membrane viruses generate intracellular enveloped virions (IEVs) by enclosing themselves in membranes manufactured by the Golgi apparatus. Moreover, these intraepithelial vesicles merge with the inner cell membrane of the host cell to generate cell-associated virions (CEVs), which are then released into the extracellular spaces to manifest as extracellular enveloped virions (EEV) [32]. As shown in Figure 4, MPXV undergoes a complex replication cycle involving host cell entry, cytoplasmic replication, virion assembly, and excretion, with multiple stages offering potential antiviral drug targets (Figure 4).

## 5. Clinical Symptoms

**Figure 5:**
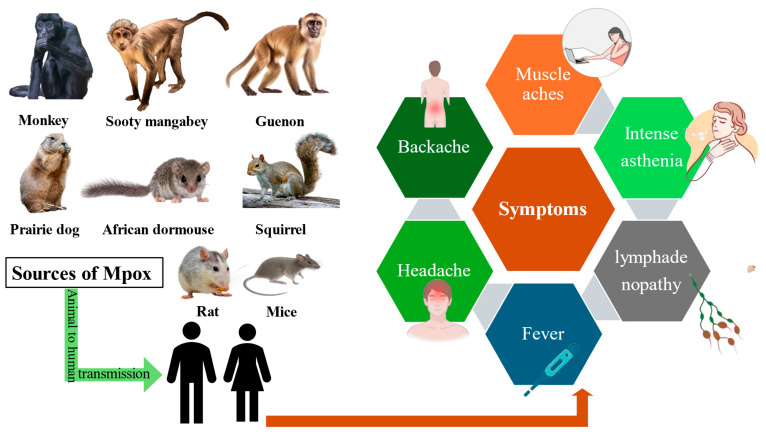
Various rodents and primates act as reservoirs for Mpox, which can transmit to humans and cause symptoms such as fever, headache, backache, and lymphadenopathy.

Mpox infection gives rise to clinical manifestations after an incubation period of 5–21 days, followed by flu-like symptoms such as fever, headache, muscle pain, fatigue, and swollen lymph nodes. Within a few days, a rash appears on the face and spreads to the hands, feet, and genitals, progressing from macules to papules, vesicles, pustules, and scabs that heal in 2–4 weeks. Most cases are mild, but severe illness can occur in children, pregnant women, and immunocompromised individuals. Complications may include bacterial infections, pneumonia, or eye damage [43]. Clade I causes more severe disease, while Clade II causes milder. In addition to this, the majority of cases that have occurred since the outbreak of 2022 are males (98%) and were engaged in homosexual activities [30]. The most frequent signs and severe health repercussions include respiratory distress and bronchopneumonia, sepsis, ulcers in the mouth, throat, and gastrointestinal tract, fever, superinfection of the skin, inflammation and lymphadenopathy, ocular infection, and skin scarring, cellulitis, and skin lesions. Clinical manifestations that persist for a period of two to four weeks may manifest unexpectedly and progress gradually [44]. Several rodents and non-human primates serve as natural reservoirs for Mpox, which can be transmitted to humans, resulting in systemic symptoms including fever and muscle aches (Figure 5).

**Figure 6:**
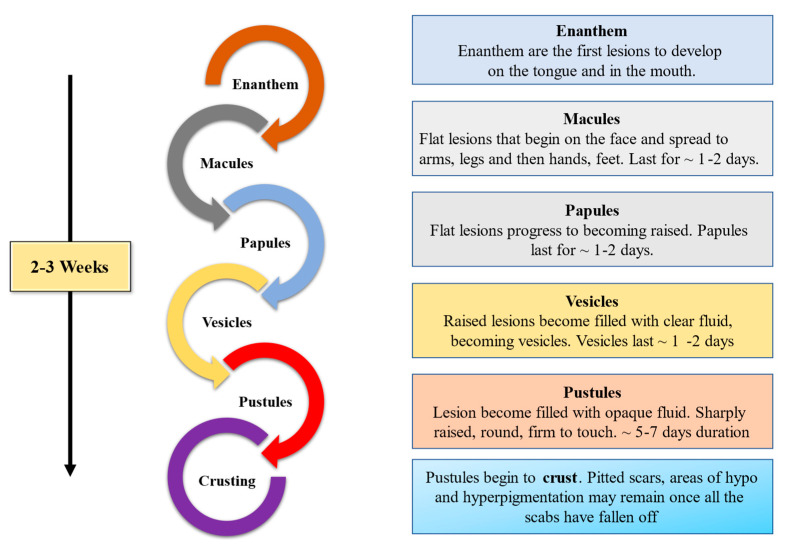
Stages of the vesiculo-pustular rash in Mpox patients. The figure illustrates the sequential progression of skin lesions from macules to papules, vesicles, pustules, and finally scab formation. These characteristic lesions appear simultaneously in the same developmental stage within a localized area, aiding in the clinical diagnosis of Mpox infection.

After gaining entry into the cells via the respiratory mucosa, MPXV, usually takes between seven and twenty-one days to produce the clinical symptoms [33]. During the 2003 U.S. outbreak, a child exposed to an infected prairie dog developed widespread flat red macules on the chest, extremities, palms, and soles, which later spread to the face and oral cavity. The child subsequently experienced fever, headache, muscle aches, fatigue, chills, lymphadenopathy, and progressive difficulty swallowing, breathing, vomiting, and eating over the next five days. A small number of lesions were discovered in the external genital region, which were shielded by the vulval mucosa [45]. In the same year, a youngster who was six years old and with severe encephalitis, along with common symptoms, was brought to the hospital [46]. As shown in Figure 6, Mpox lesions typically progress through distinct stages; macular, papular, vesicular, pustular, and scab within a defined time frame (Figure 6).

## 6. Reservoir

In addition to monkeys, the MPXV is capable of infecting various animals; specifically, small mammals make up the majority of the infected animals [47]. The illness or antibodies to the MPXV have been found in several other species, including monkeys, rats, prairie dogs, and squirrels [48]. Graphiurus species and cricetomys had low seroprevalence rates, according to research that was conducted on rats in Ghana [49]. Funisciurus species have been shown to have greater seroprevalence rates. There is a possibility that the Funisciurus species are among the natural reservoirs of the MPXV. Although no material can be curtained and considered conclusive as the natural reservoir of the MPXV. The lack of viral isolates from animal species, even from those that were serologically positive [48], makes the process challenging. Based on the findings of several investigation reports, it is suggested that small mammals contain MPXV naturally. The worldwide trade in exotic pets is considered to be the potential contributor to the transmission of MPXV. An epidemic that occurred in the United States in 2003 was linked to interaction with prairie dogs that were afflicted with the disease. The transmission of illness from animals to people has been linked to the exposure of humans to the excretions and secretions of an infected animal [50]. Prairie dogs themselves had contracted the disease as a result of coming into contact with rodents that had been introduced from Africa [51]. In the process of locating an animal reservoir, the use of serology to identify orthopoxvirus presents several interesting challenges. Since immunoglobulin M and immunoglobulin G are also generated with other Orthopoxvirus species, serology testing for these antibodies does not provide results that are unique to the MPXV [52]. There exists a definite potential that serology tests are discovering orthopoxviruses that are not the MPXV. Since relatively few viruses have been isolated from animal species, this makes the analysis of determining the natural reservoir of the MPXV more complicated.

## 7. Diagnostic Assays

It is difficult to diagnose the MPXV by relying just on clinical symptoms; hence, molecular assays and testing of patient material are beneficial and significantly essential for carrying out case confirmation. It is possible to identify Mpox or orthopoxvirus in clinical specimens obtained from patients by using a variety of diagnostic procedures [53]. The collection and examination of samples must be carried out in accordance with the established guidelines for standard precautions.

The diagnosis of MPXV may be accomplished using techniques such as viral culture, imaging using electron microscopy, immunohistochemistry, anti-orthopoxvirus IgG and IgM, and real-time polymerase chain reaction (RT-PCR) [24]. Polymerase chain reaction (PCR) may be used on its own or in combination with sequencing [54], both of these options are accessible. To get the most accurate findings, it is recommended to combine these tests with clinical and epidemiological data, such as a patient’s vaccination history [29].

PCR is applied to the diagnosis of Mpox; however, there is a dire need for technologically advanced equipment and facilities, skilled healthcare HR and sustainable public–private partnership to achieve the success model for early detection of Mpox infection. These requirements limit its application in low-resource environments. CRISPR-based tests and isothermal amplification techniques like LAMP and RPA have faster outcomes, require fewer instruments, and have potential for point-of-care treatment [55,56]. Latest research has documented that these platforms have the potential to be as sensitive and specific as PCR under laboratory conditions. But they still require the large-scale field validation, reduced costs, constant reagents, and government consent. When further optimized and incorporated into centralized testing systems, this would significantly enhance the Mpox surveillance and outbreak response in areas with limited resources [57,58,59].

Listed below is a list of diagnostic methods, as well as presented in Table 4, that may be used to differentiate clinical specimens from either orthopoxvirus or MPXV:

- Viral culture and isolation use patient specimens to grow and identify live viruses, enabling accurate species classification. The process is time-consuming and requires skilled personnel to prevent contamination [29].
- Electron microscopy detects viral particles in samples like scabs or fluids, revealing the brick-shaped structure typical of poxviruses. It must be performed in a well-equipped laboratory by trained experts [29].
- Immunohistochemistry identifies orthopoxvirus-specific antigens in biopsy samples, helping distinguish MPXV from other agents. The test requires specialized laboratories and expert personnel [29,60].
- The serology test assesses antibodies against orthopoxviruses using immunofluorescences or neutralization tests [61]. This test is known as the anti-orthopoxvirus IgG and IgM test. There is a possibility that vaccination might interfere with serologic testing, which is why the WHO does not suggest using antibody testing alone for the diagnosis of MPXV [54].
- Conventional PCR and real-time PCR assays were designed to precisely target the DNA of the MPXV by exploiting lesion material from active patients. These concerns about contamination are warranted since it is a procedure that is very sensitive [32].

In contrast, these examinations need expensive supplies and apparatus, and they must be carried out by qualified technicians [32]. Investigations are now being conducted to discover molecular approaches that are quick, simple, and accurate for the diagnosis of MPXV. For example, Chen et al. developed a portable CRISPR–Cas-based system for the detection of MPXV using just the naked eye [62]. Singh et al. recently published a report in which they outlined a strategy that targets a single nucleotide polymorphism (SNP) on the *polA* gene in order to differentiate MPXV from other orthopoxviruses that are linked to it [63]. The RPA amplification, which is then followed by the CRISPR-Cas12a, provides the basis for the diagnosis. These approaches have a high level of sensitivity, which makes them potentially suitable for application in point-of-care testing of MPXV.

It is central to critically examine emerging point-of-care diagnostics platforms for Mpox. Recent assays, such as CRISPR-based and isothermal amplification technology, have demonstrated good sensitivity and specificity in controlled experiments. However, the testing accuracy is determined by the type of sample, viral load, operator expertise, and environmental factors [64]. Practical performance is also based on several factors such as cost, ease of use, supply-chain-strength, and compatibility of the tests with current health systems. The adoption of these tools for large-scale surveillance should be preceded by a balanced evaluation of their accuracy of analysis and feasibility of operations, particularly in low-resource settings [59,64].

**Table 4:** Diagnostic Methods for MPXV [60,65,66].

Methods	Principle	Limitations/Requirements
PCR	Detects MPXV DNA through molecular amplification.	Requires molecular lab setup, costly reagents, and trained staff.
Viral Culture and Isolation	Cultivation of live virus to identify and characterize MPXV.	Time-consuming, needs BSL-3 lab and expert handling; contamination risk.
EM	Visualization of characteristic brick-shaped virions using negative staining.	Requires electron microscope and expert personnel; not species-specific.
IHC	Detects orthopoxvirus-specific antigens in tissues using labeled antibodies.	Not specific to MPXV; it needs skilled pathologists and quality reagents.
Serology (IgM/IgG)	Detects anti-orthopoxvirus antibodies via ELISA, immunofluorescence, or neutralization tests.	Vaccination may interfere; WHO does not recommend serology alone for diagnosis.
ELISA	Detects viral antigens using enzyme-linked immunoassay or lateral flow kits.	Lower sensitivity may cross-react with other orthopoxviruses.
NGS	Determines the full genome sequence for variant identification and epidemiology.	Expensive; needs advanced bioinformatics and equipment.
LAMP	Detects MPXV DNA at a constant temperature without thermocycler.	Slightly less sensitive than PCR; risk of contamination if mishandled.
LFIA	Detects orthopoxvirus antigens using portable test strips.	Low sensitivity and specificity; confirmatory PCR needed.

Lateral Flow Immunoassay (LFIA), Loop-Mediated Isothermal Amplification (LAMP), Next-Generation Sequencing (NGS), Immunohistochemistry (IHC), Electron Microscopy (EM).

## 8. Treatment

The vaccine against the vaccinia virus has historically been able to protect against Mpox; however, ever since the smallpox epidemic was eradicated, this form of vaccination has been unfollowed. Consequently, the treatment alternatives available to people who are already infected are of utmost significance [67]. There is not a single antiviral medication that has been licensed by the Food and Drug Administration (FDA) of the United States of America that is especially designed to target the MPXV. Nevertheless, in laboratory trials, it has been shown that alternative drugs, such as tecovirimat (TPOXX/ST-246) and brincidofovir, which are both effective against smallpox, as well as cidofovir, an antiviral that is authorized to combat CMV, are also effective against orthopoxviruses. Tecovirimat (TPOXX) was granted approval by the FDA of the United States of America in 2018 for the treatment of smallpox in both adults and children [68]. This medicine is effective because it inhibits the correct functioning of VP37, which is a protein that wraps around the viral envelope, and it also effectively blocks viral reproduction and release. Under an expanded-access investigational new drug procedure (EA-IND), it is presently offered in the United States of America at no cost [69]. The FDA announced in June 2021 that brincidofovir may be used to treat smallpox in both adults and children. This prodrug of cidofovir is composed of a lipid conjugate, and it is transformed into cidofovir diphosphate (CDP) inside the cells. This compound inhibits the viral DNA polymerase, which ultimately leads to the cessation of the virus’s reproduction [70]. Table 5 summarizes the currently evaluated antiviral agents for MPXV, highlighting their mechanisms, efficacy, and clinical application.

Emerging clinical data indicate that Tecovirimat (TPOXX) is widely applied for Mpox treatment, and its therapeutic benefit remains uncertain. This confirmation was derived mainly from observational cohorts and compassionate reports, which cannot reliably establish efficacy. Recently completed randomized controlled trials provide clearer insight. The PALM007 double-blind RCT conducted in the Democratic Republic of the Congo showed that Tecovirimat was safe but did not significantly shorten the time to lesion resolution compared with the placebo [71]. Similarly, interim analyses from the multicountry STOMP randomized, placebo-controlled trial found no significant improvement in lesion healing, symptom duration, or pain relief, leading to early closure of the randomized arm [72]. These findings are consistent with current WHO, CDC, and EMA assessments, all of which emphasize that well-powered, rigorously designed multicenter RCTs are urgently needed to determine Tecovirimat’s true clinical efficacy, optimal dosing, treatment timing, and potential for resistance. Robust RCT data are also lacking for Brincidofovir, Cidofovir, and VIGIV, underscoring the need for further controlled clinical studies [73,74].

The strong pre-clinical efficacy and favorable safety profile of Tecovirimat have reported to be used as treatment against Mpox under emergency access and compassionate-use programs. Although recent randomized controlled trials have documented limited clinical benefit in shortening illness in unusual cases of Mpox without complications or in reducing the severity of symptoms [75]. This discrepancy is likely indicative of the differences between controlled trial subjects and those in field use, including the severity of disease, immunological condition, and study outcomes. Recent findings suggest the potential continued applicability of tecovirimat to high-risk patients, such as immunocompromised individuals. However, routine usage should be cautious in the general use of the drug in mild cases, and further specific targeted trials are warranted [76,77].

To date, there are certain limitations in treating Mpox, such as unauthentic, insufficient clinical data, antiviral resistance, mutation, and prompt effective treatment availability. On top of this, polytherapy has not been authentically reported against Mpox infection. Mutations in viral target genes have caused tecovirimat resistance in laboratory settings and in animal models; however, it has been demonstrated that rare clinically significant resistance cases have been confirmed in humans. Combination with antiviral therapy has been suggested to minimize the development of resistance and enhance the results of patients with severe or immunocompromised diseases [78,79].

**Table 5:** Summary of Antiviral Agents and Therapeutic Candidates Against MPXV [80,81,82,83].

Antiviral Agent	Target Mechanism	Evidence Level	Current Clinical Status	Key Outcomes	Limitations
Tecovirimat (TPOXX, ST-246)	Inhibits VP37 (F13L) envelope protein, blocking viral egress	In vitro and animal studies; observational human data; RCTs	FDA-approved for smallpox; evaluated for Mpox in randomized trials	Favorable safety profile; strong antiviral activity in preclinical models; no consistent clinical benefit demonstrated in uncomplicated Mpox in RCTs	Limited efficacy in mild disease; lack of robust RCT benefit; potential for resistance with widespread use
Brincidofovir (CMX001)	DNA polymerase inhibitor (lipid conjugate of cidofovir)	In vitro, small case series	Phase II clinical evaluation	Shortened viral shedding in some patients	GI adverse effects; hepatotoxicity
Cidofovir	Viral DNA polymerase inhibitor	Animal, in vitro	Emergency use	Potent antiviral activity	Nephrotoxicity
NIOCH-14	MPXV replication inhibitor	Pre-clinical	Experimental	Promising early efficacy	Data limited to animal models

Therapeutic outcomes summarized in Table 5 distinguish between preclinical efficacy, observational clinical use, and evidence from randomized controlled trials. In comparison to cidofovir, brincidofovir has a number of advantages, including the fact that it may be taken in tablet and liquid form. A similar mechanism of action is used by both cidofovir and brincidofovir, which is its prodrug. Although there is a lack of evidence from human subjects about the efficacy of cidofovir against Mpox, there is research conducted on animals that demonstrates its efficiency against orthopoxviruses, including cowpox, vaccinia, ectromelia, and rabbitpox [84]. It was reported by Thornhill et al. that patients from the Mpox epidemic in 2022 were being treated with cidofovir, which is only available in an injectable formulation, but has the potential for severe kidney damage [31]. To date, no ultimate effective therapy is available for MPXV, which underscores the dire need for researchers to focus on developing an effective treatment approach to combat the present epidemic. Treatment options for MPXV are represented in Table 6.

## 9. Immunity to MPXV

**Figure 7:**
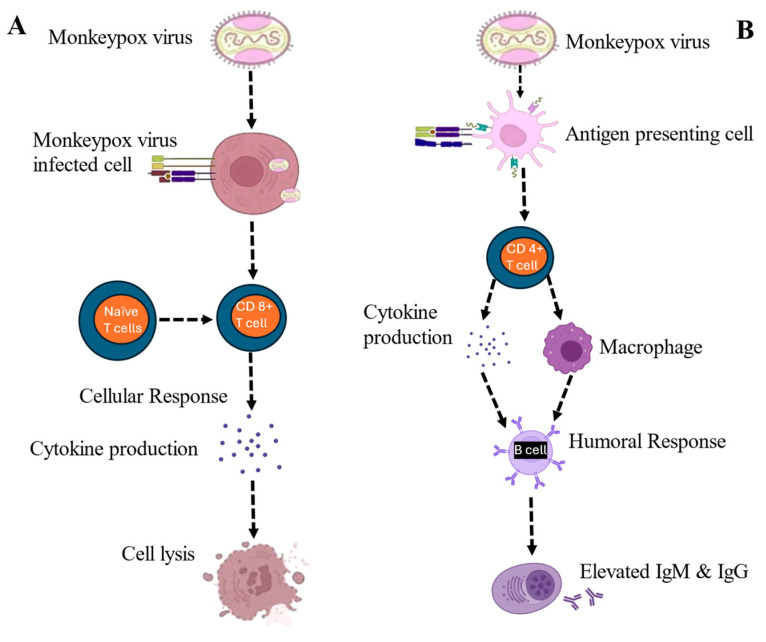
Schematic representation of the cellular and humoral immune responses to MPXV. (**A**) The cell-mediated immune response. (**B**) The humoral immune response produces IgG and IgM.

MPXV, a zoonotic pathogen of the genus Orthopoxvirus, induces complicated host immune responses, both innate and adaptive responses. These immune pathways are at the center of viral control, clinical outcome, and long-term protection after infection or vaccination. Understanding MPXV immunity can offer crucial data on correlates of protection and can be used to design next-generation antiviral vaccines and antiviral interventions. As summarized in Figure 7, the cell-mediated and humoral immune response to the MPXV infection or vaccine has been shown (Figure 7) [87].

The variation in clinical severity between MPXV Clade I and Clade II infection is probably due to different interactions between the immunomodulatory factor and host innate and adaptive immunity. Clade I viruses could have a greater ability to impair interferon signaling and antigen presentation, which activates the innate immune system later. Clade II infections tend to induce more effective immune control and mild disease [88,89].

### 9.1. Innate Immune Responses

The initial protection against MPXV infection is the innate immune system. Viral component recognition by pattern recognition receptors (PRRs) of Toll-like receptors (TLR 2, 3, 9), cytosolic DNA-sensing (cGAS, cyclic GMP-AMP synthase), and STING (stimulator of interferon genes) and RIG-I-like receptors results in intracellular signaling cascades that produce type I interferons (IFN-α/β), tumor necrosis factor-α (TNF-α), and interleukin-6. These cytokines trigger the interferon-stimulated genes (ISGs), which inhibit viral replication and stimulate the antigen presentation of dendritic cells (DCs) [90,91].

Dendritic cells and macrophages are antigen-presenting cells (APCs), which take up the MPXV antigens and move to the local lymph nodes to activate T cells. The natural killer (NK) cells are important during the initial stages of infection because they destroy infected cells and secrete IFN-γ that amplifies the immune response of the macrophage and the T cells [92]. Lack of type I interferon signaling in experimental models has a significant effect to amplify the viral load and mortality, suggesting the importance of innate immunity in its initial containment [61,93].

### 9.2. Adaptive Immune Responses

Recovery and protection over the long term are determined by the adaptive immune response. It has components of humoral and cell-mediated responses, which are synergistic [94].

#### 9.2.1. Humoral Immunity

Neutralization and prevention of reinfection do not happen without antibody-mediated immunity. Infection with MPXV or vaccination with vaccinia-based vaccines causes vigorous stimulation and differentiation of B cells into plasma cells producing virus-specific IgM and IgG antibodies. Passive immunization against conserved surface antigens, including A27L, A33R, B5R, and H3L proteins, plays a vital role in viral penetration, release, and membrane fusion, neutralization [95]. Early IgM is developed in the first 7–10 days after infection, and IgG antibodies develop at approximately 2–3 weeks and may last for a lifetime. The antibodies inhibit viral adhesion, complement activation, and opsonization and antibody-dependent cytotoxicity (ADDC) [96,97].

Serological studies of serum following smallpox vaccination in humans have shown that neutralizing antibodies are long-lived and that titers of protective levels are still detectable over years post-vaccination. Non-human primate (NHP) challenge models have also verified that passive transfer of immune sera of vaccinated donors prevents lethal MPXV challenge, and this demonstrates the protective role of humoral immunity [42,98].

#### 9.2.2. Cell-Mediated Immunity

T cell response plays a central role in the suppression of MPXV replication and infection clearance. The CD4+ T helper (Th) cells promote B cell maturation and isotype change, producing such cytokines as IFN-γ, IL-2, and TNF-α, which promote macrophage activation and cytotoxicity. The cytotoxic T lymphocytes (CTLs) or the CD8+ cytotoxic T cells can recognize the viral peptides that are presented by the MHC class I molecules on infected cells and induce apoptosis through perforin and granzyme [99,100]. The significance of CD8+ T cell responses correlates strongly with the clearance of viruses and survival of infection, as shown by animal models and human vaccine trials [101].

#### 9.2.3. Memory Immunity

When a person is infected or vaccinated, long-lived memory B and T cells are produced, which ensure rapid and vigorous responses of recall when re-exposed. Research on smallpox vaccination provides evidence that memory T cells last for several years, and the antibody-mediated immunity lasts over three decades. This long-term immunological memory is the reason why older populations that were previously vaccinated have a lesser severity of MPXV disease and shorter illness duration than those who were not vaccinated [42,99,102].

It has been demonstrated that both neutralizing antibodies and T-cell-mediated responses contribute to protection against Mpox. Humoral immunity prevents infections, whereas cellular immunity causes the elimination of the virus and the attenuation of the disease. The proportion of significance, stability, and threshold of these immune components remains unclear in various populations and viral clades. This gap has implications for the next-generation vaccine design and the importance of platforms that trigger strong, sustained, and balanced humoral and cellular immune responses, and clinical experiments to determine dependable protection markers [89,103].

The sustained immunity against MPXV is probably mediated by long-term memory B cells and T cells. Upon re-exposure to antigen or vaccinations, memory B cells induce the rapid production of antibody-secreting plasma cells and contribute to rapid neutralization at a time when the antibodies in circulation are diminishing. The presence of virus-specific CD4+ and CD8+ memory T cells restricts replication and enhances the clearance through enhanced cytotoxic activity, and supports antibody production [99,104].

#### 9.2.4. Mechanisms of Immune Evasion of MPXV

MPXV has comparable immune evasion methods as compared to other orthopoxviruses: it inhibits interferon signaling, modulates the response of inflammatory cytokines, and interferes with antigen presentation. The genomic and functional studies indicate that MPXV does not have or exhibits low activity of specific genes that are present in more virulent orthopoxviruses [42]. This decreased activity demonstrates the less pathogenic and clinical severity of MPXV. Such differences are principal to therapy: although broad-spectrum antivirals and vaccines are effective against orthopox viruses, there may be certain differences in how therapies are affected by the specific immune interactions of MPXV, the progression of the disease, and the development of specific immunomodulatory or antiviral therapy [90,105].

In the case of infection, MPXV coordinates several processes of immune evasion that influence viral replication and host response. At the early stages, production and signaling of type I interferon are inhibited by viral proteins, preventing the induction of interferon-stimulated genes, enabling successful replication at the point of entry. At the same time, MPXV releases pro-inflammatory cytokine-binding or neutralizing proteins, which suppress the immune cell recruitment and suppress early inflammation [90]. This natural immunity suppression enhances early viral growth, postpones the presentation of antigens, slows the development and activation of adaptive immunity, in particular, the activation of the CD8 + T-cells. With further replication of the virus, weakening or partial overcoming of the immune evasion, the adaptive immunity starts to purge the virus. These time-dependent interactions indicate immune evasion as an important determinant in MPXV pathogenesis and emphasize the importance of therapeutic approaches that would restore native signaling [90,94].

**Figure 8:**
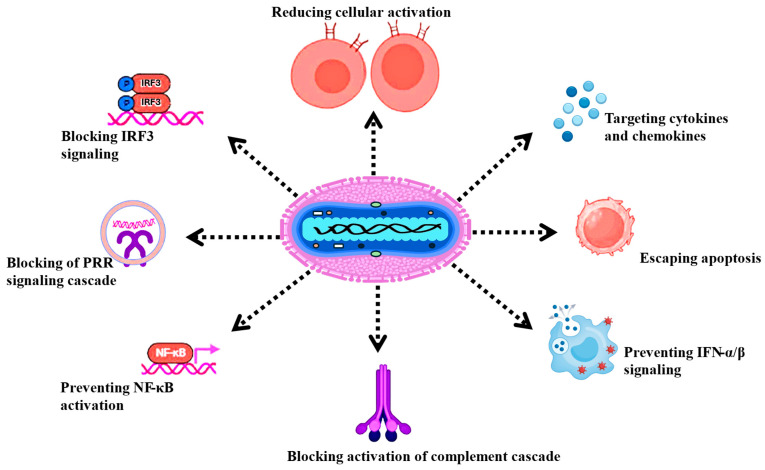
Immune evasion strategy by MPXV.

Like other orthopoxviruses, MPXV expresses a large number of proteins that regulate or evade the response of the host immune system, as shown in Figure 8.

#### 9.2.5. Cross-Protection and Immunity Caused by Vaccines

Previous studies suggest that smallpox eradication and vaccinia-based vaccines are associated with some 85% cross-protection against MPXV. Vaccinated persons who were vaccinated before 1980 present milder disease after exposure, and they have quicker viral clearance [106]. The new non-replicable types of vaccines, like MVA-BN (Jynneos/Imvamune/Imvanex) produce humoral and cell-mediated immunity with enhanced safety in immunocompromised persons [107]. Replication-competent vaccines (e.g., ACAM2000) are safe, but they induce significant immune responses and are associated with adverse reactions in the immunocompromised or pregnant populations. The efficacy of vaccines will focus on the production of balanced antibody and T cell responses that will neutralize both clade I and II MPXV strains [108,109].

Preexisting immunity due to earlier smallpox vaccination influences the progression and clinical severity of Mpox, particularly in older individuals. The vaccinated individuals have prolonged B and T-cell memory against conserved orthopoxvirus antigens, enabling rapid activation upon exposure to MPXV [110]. This latent immunity is associated with less viral loads, milder symptoms, fewer lesions, and quicker recovery than individuals who are not vaccinated before. These findings highlight the role of immune memory in determining outcomes, providing a public health context for risk assessment, prioritizing vaccines, and bolstering policies during present and future Mpox outbreaks [111].

Replicating vaccines like ACAM2000 and LC16m8 promote robust antigen presentation, produce strong cytotoxic T-cell responses mediated by CD8+ cells, and long-lasting memory T cells and neutralizing antibodies. Their replicative aspect poses a reactogenicity and safety concern in immunocompromised individuals [112]. MVA-BN (JYNNEOS) is non-replicating and induces immunity primarily through neutralizing antibodies and CD4+/CD8+ T cells, without replication, and has a superior safety profile. Even though MVA-BN induces poorer antibody and cellular responses than the replicating vaccines, the resultant combination of humoral and cellular immunity is suggested to produce sufficient cross-protection against MPXV. These differences can be understood to maximize the benefits of choosing vaccines, boosters, and designs of next-generation vaccines, which may confer maximum protection and yet be safe [113,114].

## 10. Vaccines for the MPXV

**Figure 9:**
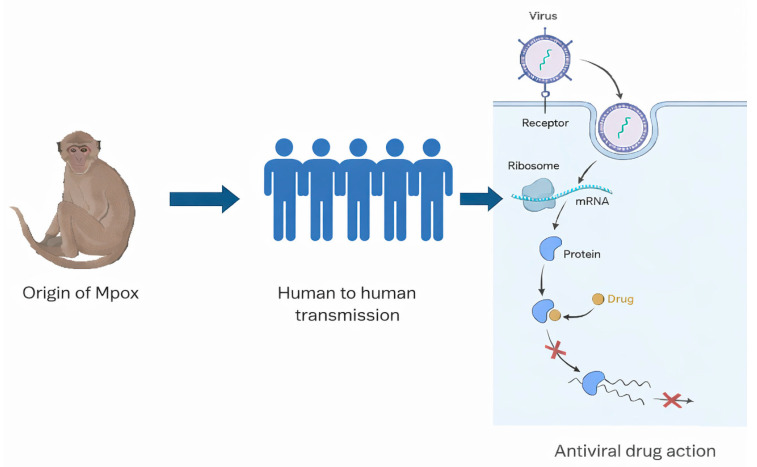
A schematic illustration shows the zoonotic origin of the virus from monkeys, human-to-human transmission, and the role of vaccination in prevention. The diagram on the right depicts the antiviral mechanism, where drugs inhibit viral replication by interfering with mRNA translation and protein synthesis within host cells.

Several measures are required to protect individuals from MPXV infection and to prevent further transmission. Although vaccines are the most effective tools for achieving this, no vaccine has yet been specifically developed for MPXV [65,115]. Although no vaccine is specifically designed for MPXV, several smallpox vaccines offer effective cross-protection, as shown in Figure 9. The ACAM2000 (a second-generation smallpox vaccine) is FDA-approved and stockpiled for emergency use [116]. The JYNNEOS (MVA-BN) vaccine, developed by Bavarian Nordic, is a third-generation, non-replicating vaccine requiring two doses given 18 days apart, providing strong immunity and a safer profile [117]. Another third-generation vaccine, LC16, developed by KM Biologics, has shown good safety and immune response and is approved in Japan and the United States for smallpox and Mpox prevention [118,119]. This was because the protective effects shown in animal research were confirmed to be safe, and the immunizing activity observed in human trials was confirmed [120]. Details of ongoing and recent therapeutic and vaccine clinical trials for MPXV are summarized in Table 7.

### 10.1. Smallpox Vaccines and Cross-Protection

Smallpox is a contagious disease eradicated in 1980 as a result of a global vaccination campaign applying vaccinia virus vaccines. While there were no specific therapies for the MPXV, information from Zaire in the 1980s indicated effective cross-protection from MPXV exposure. During the major global smallpox outbreak in the year 2022, about one million doses of modified smallpox vaccines were given as pre- and post-exposure prophylaxis to the population at risk of MPXV [71,123]. The secondary MPXV infection rates in unvaccinated close contacts and those who had previously had a smallpox vaccination were compared in two separate investigations conducted in Zaire in the 1980s. The findings indicated that the rate of secondary infection incidents was 7.4% in people without previous records of smallpox immunization and 1.1% in those with a history of smallpox vaccination. Following research, prior immunizations offered protection against Mpox in close, face-to-face contact of between 85.1% and 87.1% [72,108].

ACAM2000 is a second-generation vaccine produced from a single clonal viral isolate of Dryvax, demonstrated to have decreased neurovirulence in animal studies. Rather than using the previous method of scarification on the flanks of calves (Bos taurus), tissue culture is employed for their propagation. Clinical trials exhibited a similar safety profile to Dryvax, and immunogenicity testing proved to be non-inferior to Dryvax [73]. Administration of ACAM2000 is specifically discouraged in any patient with immunosuppression due to the significant risks associated with the vaccine, which include myopericarditis, especially in those who have not had smallpox. Many virus passages in primary cell cultures or eggs are employed as the virus attenuation approach to generate new vaccines that are suitable for further attenuation [74].

The ACAM2000 vaccine, licensed in the United States by the US-FDA in 2015, is a second-generation post-exposure prophylactic for smallpox, making it the only vaccine for Mpox from 2015 to 2019. Due to the similarities between smallpox and Mpox, vaccines against smallpox, such as ACAM2000, offer a moderate level of protection against Mpox, either prior to or following virus exposure. A 1980 research study carried out in the Democratic Republic of the Congo revealed that vaccinations based on the vaccinia virus produced an 85% protection against mpox; the report did not, however, indicate which vaccine was administered to the recruited individuals [124,125]. Constitutional symptoms such as fever, malaise, headache, myalgia, and lymphadenitis are among the side effects of ACAM2000. Rare severe side effects, such as skin infections, encephalopathy, encephalomyelitis, progressive vaccinia, erythema multiform, myocarditis, pericarditis, and post-vaccination encephalitis, have been reported [126].

### 10.2. Advancements in Mpox Vaccines

Innovations in the Mpox vaccine focus on safety precautions as well as increasing vaccine availability among the high-risk groups. Clinical trials conducted in 2023 found efficacy in lowering the severity of the disease, preventing infections, and minimizing the rate of transmission. As for the current conditions and the spread of Mpox in many states of the world, and the possibility of the re-establishment of smallpox in connection with the use of the vaccine, it appears that it would be more advisable in the present situation to concentrate the production of the Mpox vaccine. Currently, there are many ongoing research projects to investigate and develop effective anti-Mpox vaccines [67,99].

#### 10.2.1. MVA-BN (Modified Vaccinia Ankara-Bavarian Nordic)

The live attenuated, non-replicating Modified Vaccinia Ankara-Bavarian Nordic (MVA-BN) vaccine, also marketed by the trade names Imvamune, Jynneos, and Imvanex, is a third-generation smallpox vaccine. In 2019, the FDA authorized the MVA-BN vaccine to prevent MPX and smallpox in high-risk individuals. Bavarian Nordic A/S produced the replication-competent ACAM2000^®^ vaccinia Ankara strain JYNNEOS, which is safe and effective against MPX and smallpox in adults 18 years of age and older. Patients with atopic dermatitis and HIV who live in MPX-endemic regions can also benefit from it [127,128,129]. Replication of the MVA strain was limited to avian and mammalian cell types after 570 passages in chicken embryo fibroblast cells. There are single 0.5 mL dosages of JYNNEOS, a subcutaneous injectable suspension. A person is considered completely immunized approximately two weeks following their second dose of JYNNEOS [130]. Up until now, JYNNEOS, which has received proper approval for the Mpox immunization, appears to be a safer choice. However, research is still needed to fully comprehend the vaccine’s immunogenicity as well as its safety profile. It has been reported to be safe for immunocompromised individuals, such as those with atopic dermatitis, HIV patients, and recipients of transplants. Comparing the ACAM2000 vaccination to clinical trials, adverse effects such as redness, discomfort, induration, itching, sore throat, myalgia, headache, chills, and nausea were shown to be more tolerable and less severe for the injection site [131]. A live, attenuated strain of the vaccinia virus that is incapable of replicating in human cells is used in the MVA-BN smallpox vaccination. The 2022 MPXV strain (MPXV-2022) and the MVA-BN virus used in this vaccination have highly similar genomes, where ≥93% amino acid sequence similarity is shown by the protective antigens A29, A35, B6, M1, H3, and I1 [107]. The MVA–BN vaccine has been recommended against Mpox in many countries, including in the UK, Europe, and the USA. Additional safety monitoring data have also been collected in the US during the 2022 Mpox outbreak, which received nearly one million doses [71,122].

#### 10.2.2. LC16m8 Vaccine

Early in the 1970s, the temperature-sensitive and low-virulence strain LC16mO from the original Lister strain was passaged several times in cell culture to produce LC16m8, an intensely attenuated strain of virus vaccine. LC16m8 produces smaller plaques compared to Lister in the chicken chorioallantois membrane. LC16m8 has temperature-dependent characteristics whereby it does not efficiently grow in primary rabbit kidney (PRK) cells at 41 °C, unlike Lister [132]. According to the studies, LC16m8 has an almost negligible level of neurovirulence in all animal models. In Japan, over 100,000 individuals were vaccinated with LC16m8 against Mpox, and it was noted that no LC16m8-related adverse events, including severe reactions or fatalities, were observed. The investigations indicate that LC16m8 is less toxic than the currently used virus vaccines extracted from bovine skin. Therefore, LC16m8 can be further studied as a candidate for the new generation of antiviral vaccines that can replace existing virus vaccines [133,134].

Japan has already registered LC16m8 because of its high immunogenicity and safety profile. It is interesting to note that although the vaccine was approved in the 1970s, it has never been utilized in Japan’s campaign to eradicate smallpox. However, when LC16m8 manufacture was resumed in Japan in 2002, its safety and efficacy were re-investigated. The LC16m8 vaccine has undergone animal testing, and the fact that it has immunized more than 50,000 children and 3500 adults in Japan confirms its efficacy [135,136]. Due to its reduced adverse effects and continued efficacy against orthopoxviruses such as Mpox, LC16m8 is regarded as a safer option than ACAM2000. This vaccination frequently causes mild to moderate systemic and local adverse effects. Serious side effects such as encephalitis and symptomatic myocarditis were not reported in clinical trials or cohort studies. Preclinical evidence suggested the administration of the LC16m8 vaccine in individuals with atopic dermatitis and weakened immune systems; however, mass vaccination is not advised in these patients [137,138]. The transmission dynamics, vaccination, and antiviral inhibition of Mpox replication are illustrated schematically (Figure 9).

#### 10.2.3. DNA and Protein Subunit Vaccines

Scientists are currently focused on investigating functional DNA vaccines and protein subunit vaccines against Mpox, which are designed to induce specific immune responses by presenting viral proteins and abandoning the utilization of live attenuated viruses as vaccine vectors [139]. A study was conducted to determine the efficacy of the subunit recombinant vaccine against Mpox in an animal model. The results of the study showed that a combination of subunit protein and DNA vaccines provided good protection against Mpox by boosting antibody titers, immunological responses, and stopping the transmission of the virus [140]. Investigation of vaccines based on genetic code and protein scaffold is currently in progress, and it is the dawn of the fourth-generation vaccines. In a preventative nonhuman primate study, they were able to achieve sterile protection with ectodomains of protein A33 and B5 from mature MPXV, Alhydrogel, and CpG adjuvants. A study’s result indicated that rhesus macaques are better protected from severe Mpox infection when vaccinated using a recombinant vaccination mode (DNA plus proteins), which could be useful for screening larger populations and providing safe baseline poxvirus immunity [141]. Table 8 provides an overview of existing and investigational vaccines against the MPXV, highlighting their platforms, immunogenicity, and clinical development stages.

There is a lack of data on the efficacy of smallpox-derived vaccines against several clades of MPXV, particularly amongst immunocompromised individuals. Non-replicating vaccine (MVA-BN, JYNNEOS/Imvanex/Imvamune) is preferable since it has managed to produce a safety profile and generates both humoral and cellular responses; the real-life effectiveness against Clade I vs. Clade II MPXV is not yet completely addressed. Replication-competent ACAM2000 and LC16m8 are highly immunogenic and cross-protective but may lead to higher risks of adverse events; hence, not recommended for usage in immunocompromised individuals [114,144]. Table 9 represents a comparison of different Mpox vaccines.

## 11. Scenario of Mpox in Asia, Including India and Pakistan

In the recent outbreaks, the WHO has documented the first cases of Mpox in Europe, with no evidence linking them to West and Central Africa in terms of epidemiology. Endemic Mpox has previously been reported in Cameroon, Central African Republic, Cote d’Ivoire, Congo, Gabon, Liberia, Nigeria, Republic of Congo, and Sierra Leone. The rest of the countries in Asia except Israel had only a few cases of Mpox which included South East and Middle East region with 16 cases in the United Arab Emirate, 15 in Singapore, six in India, five in Saudi Arabia, four in Thailand, two in Qatar, two in Taiwan, two in Japan, one in South Korea and one in the Philippines [145].

Till May 31, 2022, India had not reported a case of Mpox. Thus, with the first case reported in India, there is a gradual increase in cases across different regions in India [146]. By October 2025, Asia had registered an estimated more than 4000 confirmed cases, and the region had reported a few cases of deaths. The first death from India was reported in a 22-year-old man from Kerala who had a travel history in the UAE. There were multiple suspected cases reported from UP, Bihar, and Telangana as well; however, two labs confirmed Mpox cases from Delhi and Kerala only, respectively [147]. The country has since reported approximately 40–50 confirmed cases and one death, which were mainly in Kerala, Delhi, and Maharashtra. The majority of the infection has been mild and self-limiting, with minimal community transmission [148]. Medical supervision is required in severe or high-risk patients who take tecovirimat, an antiviral medication, used in the treatment of infections caused by orthopoxvirus [149]. To increase accessibility and shorten testing times, confirmatory testing was first conducted at the National Institute of Virology in Pune. It was then launched at AIIMS in Delhi and is currently offered in 15 distinct specialist laboratories [150,151].

The first mpox case in Pakistan was reported in April 2024 and involved travelers who returned to the country after staying in the Gulf. The Mpox infection disease was reported to range between 20 and 25, with no deaths so far, and many cases were recorded up to October 2025. The incidence of Mpox infection cases has been observed to occur with no significant long-lasting immunity and community-based transmission [152]. Active surveillance, swift diagnostic procedures, and the increased testing capacity for Mpox infection by the National Institute of Health (NIH), Islamabad, produced promising outcomes as far as the disease spread is concerned. This practice has been extended to provincial laboratories in Punjab, Sindh, and Khyber Pakhtunkhwa. The government has also strengthened its screening of airports and borders, set quarantine centers, and provided campaigns to the masses about their health to halt local spreading [153,154]. In the context of the Mpox outbreak, Pakistan has asked authorities for expedited traveler screening. At Karachi airport, medical professionals are examining passengers and verifying suspicions with scanning devices. Airports may have different processes, depending on available resources. Airports are equipped with isolation units, logistics assistance, and skilled staff [152]. To stop the Mpox outbreak, the Pakistani government is extending surveillance, which means border health services and health authorities need to be extremely vigilant. The government will screen passengers of African descent in accordance with international health regulations and WHO guidelines [153,155].

Mpox cases have also been reported in other Asian countries, with Thailand, Japan, South Korea, China, and Singapore having recorded cases, with travelers being the main victims. Localized outbreaks in Thailand and Singapore were small, and China and Japan have been largely involved in imported cases [156]. The Middle East region, especially the UAE, Saudi Arabia, and Qatar, is still playing the role of a regional source of imported infections into South and Southeast Asia. Altogether, the Mpox situation in Asia is controlled, and the level of transmission is low, and the number of deaths is low. The majority of the infections are not through community-wide spread, but travel internationally [157]. Nevertheless, the lack of vaccination, the large population density, and the constant movement between these countries are existing challenges. The increased vigilance of the laboratory resources and social health can continue to be essential in avoiding future outbreaks in the region [158].

## 12. Future Perspective

Current research on MPXV faces several challenges. There are still no controlled clinical trials confirming the efficacy of available antivirals. Genomic surveillance in endemic African regions remains limited, restricting early detection of new variants. Long-term vaccine efficacy and safety data are also lacking, particularly in high-risk groups. Future studies should explore next-generation vaccine platforms, such as mRNA-based approaches, to improve protection. In addition, adopting a One Health framework is vital to monitor animal reservoirs and prevent future zoonotic spillovers. Future efforts against Mpox should focus on developing safer, more accessible, and next-generation vaccines that offer stronger and longer-lasting protection. As indicated in Table 10, significant gaps persist in understanding mpox ecology, vaccine development, and therapeutic strategies. While MVA-BN (JYNNEOS) and ACAM2000 provide some defense, improved mucosal or intranasal vaccines could better block infection at entry sites. Enhancing antiviral therapies like Tecovirimat and Brincidofovir, strengthening public health systems, ensuring vaccine equity, and deepening research on animal reservoirs and transmission are equally vital. Together, these advances will help make Mpox a fully controllable and preventable disease worldwide. To lower the rate of transmission and provide medical assistance, authorized organizations, research institutions, and governments should come to an agreement on early diagnostic signals. First and foremost, the development of various strategies for immunization campaigns. For this reason, it is necessary to conduct a wide variety of investigations and conduct more research to stop future potential hazards to public health and global issues [40,159].

In genomic research of MPXV isolates following the 2022 outbreaks, several nucleotide replacements were detected, some of which are associated with APOBEC3 editing. It remains unclear which functional effect of these changes influences viral fitness, transmissibility, immune evasion, and antiviral susceptibility [160,161]. At present, it is not clear whether these variants play an important role in lowering vaccine-induced protection or provide resistance to approved antivirals such as tecovirimat. Long-term functional genomics, phenotypic studies, and longitudinal surveillance should be conducted to determine clinical relevance [78,162].

The worldwide availability of Mpox vaccines is associated with regulatory, manufacturing, and equity challenges, particularly in Africa. The regulatory barriers are restrictions in the pathways of local approval, dependence on emergency-use approvals, and a lack of clinical evidence in local endemic populations. Manufacturing is still centralized, constrained production volume, lead times are long, and a small number of suppliers make it difficult to scale up [163,164]. Access is limited in high-burden areas by equity issues (high costs, advance purchase deals with high-income nations, poor cold-chain, and poor-quality health systems). To address these concerns, the international community should act in unison to increase regional output, harmonize the rules, invest in domestic research, and focus on equal distribution of vaccines [165]. Integrated Summary of MPXV Biology, Host Immunity, Immune Evasion, Diagnostics, Therapeutics, and Vaccines Highlighting Translational Gaps and Future Directions as summerized in (Table 11).

## 13. Conclusions

In conclusion, Mpox remains a significant global health concern due to its zoonotic origins, ability to spread beyond endemic regions, and the challenges it poses for public health systems worldwide. The recent advancements in vaccine development have offered useful instruments for the prevention of mpox. The vaccine MVA-BN (JYNNEOS/Imvanex/Imvamune) has demonstrated desirable safety and immunogenicity results and is currently the choice of both pre- and post-exposure prophylaxis. The current research on the next-generation vaccines, antivirals, and immunotherapeutic interventions is still advancing the level of preparedness against orthopoxvirus infections in the world. The global health landscape continues to evolve, and the fight against Mpox underscores the importance of proactive measures in vaccine development and the need for coordinated efforts in managing emerging infectious diseases.

According to emerging genomic surveillance, mutation signatures are present in MPXV genomes of post-2022 outbreaks that are consistent with APOBEC3-mediated cytidine deamination and suggest host-driven editing as a potential contributor that can facilitate evolution.

## Figures and Tables

**Table 1:** Global Mpox Situation: 2023 to Mid-October 2025 [7,8,9,10,11,12,13,14].

Year/Period	Confirmed Cases	Deaths	Most Affected Regions
2023 (Jan–Dec)	~9000 new cases (≈92,783 total)	171	Americas, Europe, Africa, Asia-Pacific
2024 (Jan–Dec)	~24,880 new cases (≈117,663 total)	263	Africa, Americas, Europe
2025 (Jan–Aug)	38,671	163	Africa (>90% of cases), Americas, Europe
2025 (14 Sep–19 Oct)	2862 new cases	17	Africa (17 countries with active transmission); sporadic cases in Europe and Asia
Global Total ≈41,500 confirmed cases in 2025 (cumulative since 2022 ≈ 160,000+)		≥180 deaths in 2025 (cumulative since 2022 ≈ ≥300) and top 5 affected countries are DR Congo, Nigeria, Cameroon, Republic of Congo, Brazil.	

Tabulated data were confirmed and laboratory-verified cases published by WHO/PAHO/ECDC up to 30 October 2025. No projections or modeled estimates were applied. Year 2025 values are preliminary according to the WHO set standards.

**Table 2:** Comparison Between Mpox, Smallpox, and Chickenpox [24,25].

Feature	Mpox	Smallpox	Chickenpox
Causative agent	MPXV (Orthopoxvirus)	Variola virus (Orthopoxvirus)	Varicella-zoster virus (Herpesviridae)
Reservoir	Rodents, primates	Humans only	Humans only
Rash distribution	Face, palms, soles, trunk	Face, palms, soles, trunk	Mostly trunk and face
Lesion progression	Synchronous (same stage)	Synchronous	Asynchronous (various stages)
Mortality rate	1–10% (higher in Congo Basin clade)	30% (eradicated)	<0.1%
Human-to-human transmission	Limited	High	High

**Table 3:** Classification and Genomic Features of MPXV [32].

Feature	Description
Family	Poxviridae
Genus	Orthopoxvirus
Genome type	Linear double-stranded DNA
Genome size	~197 kilobase pairs
Number of genes	Approximately 190–200 open reading frames (ORFs)
Virion shape	Brick-shaped, enveloped
Replication site	Cytoplasm of host cell
Clades (lineages)	Central African (Congo Basin) and West African
Notable difference between clades	Congo Basin clade more virulent and transmissible than West African clade

**Table 6:** Treatment Options for MPXV [32,85,86].

Treatment/Drug	Type/Class	Mechanism of Action	Clinical Use/Indication
Tecovirimat (TPOXX/ST-246)	Antiviral (Orthopoxvirus inhibitor)	Inhibits VP37 viral envelope protein, preventing virus release from infected cells.	First-line treatment for severe mpox cases; approved by US FDA and EMA.
Brincidofovir (CMX001)	Antiviral (nucleotide analog)	Inhibits viral DNA polymerase, blocking viral replication.	Used for severe mpox when Tecovirimat is unavailable or ineffective.
Cidofovir (Vistide)	Antiviral (nucleotide analog)	Similar to brincidofovir; inhibits viral DNA synthesis.	Alternative for life-threatening mpox infections.
Vaccinia Immune Globulin Intravenous (VIGIV)	Passive immunotherapy	Provides orthopoxvirus-specific antibodies from vaccinated donors.	Used for severe complications or immunocompromised patients exposed to MPXV.
Supportive Care	Symptomatic/general management	Includes fluid replacement, pain relief, antipyretics, and treatment of secondary infections.	All mpox cases benefit from supportive care; essential for recovery.
Antibiotics (Secondary Infections)	Antibacterial therapy	Used to treat bacterial infections secondary to mpox lesions.	Reduces risk of sepsis and secondary complications.
Vaccines (Preventive)	Prophylactic	post-exposure prophylaxis).	MVA-BN (JYNNEOS/Imvamune/Imvanex), older vaccine, limited use.

**Table 7:** Ongoing and Recent Therapeutic/Vaccine Clinical Trials [121,122].

Study ID	Intervention	Phase	Population/Location	Key Findings/Status
NCT05534984	Tecovirimat vs. supportive care	Phase III	Multi-country (U.K., U.S., Nigeria)	Data shows faster lesion healing, reduced viral load
NCT05697132	MVA-BN booster immunogenicity	Phase II	Healthy adults, Europe	Robust neutralizing antibody titers after booster
NCT05715006	DNA vaccine (A35R + B6R)	Phase I	United States	Ongoing; safety evaluation underway
NCT05668229	LC16m8 vs. MVA-BN comparative	Phase II	Japan	Recruiting; aims to compare immunogenicity

**Table 8:** Summary of Current and Emerging Vaccines Against MPXV [89,142,143].

Vaccine (Trade Name)	Type	Target Pathogen	Approval Status	Key Findings/Features
MVA-BN (JYNNEOS/Imvamune/Imvanex)	Non-replicating Modified Vaccinia Ankara (MVA)	MPXV, Smallpox	FDA (U.S.), EMA (EU), Health Canada	High immunogenicity, safe for immunocompromised and pregnant individuals; induces strong humoral and cellular responses
ACAM2000	Replicating the vaccinia virus	Smallpox, MPXV (cross-protection)	FDA approved	Effective protection; contraindicated for immunocompromised and cardiac patients due to myocarditis risk
LC16m8	Attenuated vaccinia virus	Orthopox-viruses	Approved in Japan	Good safety profile; induces neutralizing antibodies
DNA-based candidates (e.g., pVAX-A35R, pVAX-B6R)	Experimental DNA vaccines	MPXV-specific proteins	Pre-clinical	Strong antibody and T-cell response in mice
Protein-subunit candidates (A29L, M1R, A35R)	Recombinant protein subunit	MPXV antigens	Pre-clinical	High antigenicity; potential for multivalent design

**Table 9:** Comparison of Mpox Vaccines with their type, generation, and developer.

Vaccine/ Generation	Developer/Manufacturer	Licensed Name	Key Features/Notes
Dryvax/1st	Wyeth	—	Discontinued; high adverse effects
ACAM2000/2nd	Sanofi/Emergent BioSolutions	ACAM2000	Used for smallpox; provides cross-protection
LC16m8/2nd	Japan Health Agency	—	Safer than ACAM2000
MVA-BN (JYNNEOS/IMVANEX/IMVAMUNE)/3rd	Bavarian Nordic	JYNNEOS	Non-replicating, FDA-approved for Mpox
(DNA/mRNA-based)/4th in development	Multiple research groups	—	Targeting MPXV-specific antigens (A27L, B5R)

**Table 10:** Major Knowledge Gaps and Future Directions.

Research Area	Current Limitation	Suggested Focus
Transmission dynamics	Limited data on animal reservoirs	Expand One Health surveillance
Immunological response	Incomplete understanding of long-term immunity	Study T-cell and mucosal immunity
Vaccine accessibility	Unequal global distribution	Ensure equitable access and affordability
Diagnostics	Limited field-ready kits	Develop portable PCR and antigen assays
Therapeutics	Few targeted antivirals	Explore combination therapies

**Table 11:** Integrated Summary of MPXV Biology, Host Immunity, Immune Evasion, Diagnostics, Therapeutics, and Vaccines Highlighting Translational Gaps and Future Directions.

Thematic Area	Key Elements	Key Gaps/Limitations	Future Perspectives & Translational Opportunities
MPXV Biology & Genomics	Viral structure, genome organization, Clade I vs. Clade II, post-2022 mutations	Functional impact of recent mutations unclear	Functional genomics; clade-specific virulence markers
Innate Immune Responses	IFN signaling, NK cells, cytokine responses	Limited human in vivo data; clade-level differences not fully defined	Biomarkers predicting severity; immunomodulatory therapies
Adaptive Immunity	Neutralizing antibodies, CD4^+^/CD8^+^ T cells	Correlates of protection not established	Immune profiling to guide vaccine design
Viral Immune Evasion	IFN inhibitors, cytokine decoys, antigen presentation blockade	MPXV-specific evasion strategies underexplored	Targeted antivirals disrupting immune evasion
Clinical Severity & Clades	Disease outcomes, host–virus interactions	Mechanistic links between immunity and severity unclear	Integrative immunopathogenesis studies
Diagnostics	PCR, CRISPR-based assays, LAMP/RPA	Field validation and scalability lacking	Decentralized diagnostics for low-resource settings
Antiviral Therapies	Tecovirimat, brincidofovir	Optimal timing, resistance, combination therapy unknown	Stratified treatment algorithms
Antiviral Resistance	VP37 mutations	Limited human surveillance data	Resistance monitoring frameworks
Vaccines	MVA-BN, ACAM2000, LC16m8, DNA/protein vaccines	Limited data in immunocompromised and by clade	Next-generation vaccines; durability studies
Public Health & Epidemiology	Surveillance, stigma, healthcare access	Underreporting in endemic regions	Community-engaged surveillance
Equity & Global Access	Regulation, manufacturing, distribution	Endemic regions under-served	Regional manufacturing & equitable allocation

## Data Availability

No new data were created or analyzed in this study. Data sharing is not applicable to this article.

## Abbreviations

ACAM2000	Live replicating vaccinia virus smallpox vaccine
APOBEC3	Apolipoprotein B mRNA-editing enzyme catalytic polypeptide 3
CDC	Centers for Disease Control and Prevention
CD4^+^ T cells	Cluster of Differentiation 4 positive T lymphocytes
CD8^+^ T cells	Cluster of Differentiation 8 positive T lymphocytes
CRISPR	Clustered Regularly Interspaced Short Palindromic Repeats
DNA	Deoxyribonucleic acid
dsDNA	Double-stranded deoxyribonucleic acid
ECDC	European Centre for Disease Prevention and Control
ELISA	Enzyme-linked immunosorbent assay
IFN	Interferon
IFN-I	Type I interferon
IHC	Immunohistochemistry
LC16m8	Attenuated replicating vaccinia virus vaccine strain
LAMP	Loop-mediated isothermal amplification
LMICs	Low- and middle-income countries
MHC	Major histocompatibility complex
MPXV	Monkeypox virus
Mpox	Monkeypox disease
MVA	Modified vaccinia Ankara
MVA-BN	Modified vaccinia Ankara–Bavarian Nordic
NK cells	Natural killer cells
PAHO	Pan American Health Organization
PCR	Polymerase chain reaction
POC	Point of care
RPA	Recombinase polymerase amplification
RCT	Randomized controlled trial
RNA	Ribonucleic acid
SARS-CoV-2	Severe acute respiratory syndrome coronavirus 2
STAT	Signal transducer and activator of transcription
TCR	T-cell receptor
VP37 (F13L)	Viral envelope protein targeted by tecovirimat
WHO	World Health Organization
